# Vibration Property of a Cryogenic Optical Resonator within a Pulse-Tube Cryostat

**DOI:** 10.3390/s21144696

**Published:** 2021-07-09

**Authors:** Yanxia Ye, Leilei He, Yunlong Sun, Fenglei Zhang, Zhiyuan Wang, Zehuang Lu, Jie Zhang

**Affiliations:** MOE Key Laboratory of Fundamental Physical Quantities Measurement, Hubei Key Laboratory of Gravitation and Quantum Physics, PGMF and School of Physics, Huazhong University of Science and Technology, Wuhan 430074, China; yxye@hust.edu.cn (Y.Y.); d201880036@hust.edu.cn (L.H.); yunlongsun@hust.edu.cn (Y.S.); fenglei_zhang@hust.edu.cn (F.Z.); zy_wang@hust.edu.cn (Z.W.); zehuanglu@hust.edu.cn (Z.L.)

**Keywords:** optical resonator, in situ vibration measurement, cryogenic temperature, vibration sensitivity

## Abstract

Cryogenic ultrastable laser cavities push laser stability to new levels due to their lower thermal noise limitation. Vibrational noise is one of the major obstacles to achieve a thermal-noise-limited cryogenic ultrastable laser system. Here, we carefully analyze the vibrational noise contribution to the laser frequency. We measure the vibrational noise from the top of the pulse-tube cryocooler down to the experiment space. Major differences emerge between room and cryogenic temperature operation. We cooled a homemade 6 cm sapphire optical resonator down to 3.4 K. Locking a 1064 nm laser to the resonator, we measure a frequency stability of $1.3\times 10^{-15}$. The vibration sensitivities change at different excitation frequencies. The vibrational noise analysis of the laser system paves the way for in situ accurate evaluation of vibrational noise for cryogenic systems. This may help in cryostat design and cryogenic precision measurements.

## 1. Introduction

Cryogenic-cavity-based ultrastable lasers are one of the most promising options for improving laser stability by reducing the thermal noise limit to $10^{-17}-10^{-18}$ level [1,2,3,4,5,6,7]. However, the running of cryogen-free cryocooler introduces significant vibrational noise. Vibrational noise is one of the major noises that affects the performance of cryogenic ultrastable lasers [5,6,8,9]. Most cryogen-free cryostats are already equipped with vibration suppression techniques, such as soft spring connection, helium gas buffer, a liquid/gas mixing helium zone between the cold head and the cryogenic plate, or an active vibration isolation system, etc. [10,11,12,13]. Therefore, an in situ and in-depth evaluation of the vibrational noise is helpful and meaningful for frequency noise evaluation. In addition, the evaluation of vibration noise right on the cryogenic plates are also very important for many other high precision low temperature measurement experiments, such as scanning tunneling microscopes (STM) [14,15], gravitational wave detection [16,17,18], and many other examples of fundamental research [19,20].

In this paper, we use an in situ, high resolution vibration measurement equipment (geophone) under cryogenic temperature to measure the vibration level of the sample holder of a closed-cycle cryostat. Driven by the requirement of the cryogenic ultrastable laser, we carry out vibrational measurements inside a specially designed vibration-reduction pulse-tube cryostat by using the properly re-calibrated geophones. We measure the vibrational levels both when the cryostat has just been powered on and when it is in stable operation at 3.4 K. The vibrational noise is evaluated in the frequency domain to better gauge different frequency components contributions. From the comparison of the vibrational spectra, major differences can be identified when the cryostat is in different operational stages, and vibration suppression due to the designed liquid/gas helium mixture is clearly demonstrated for the first time.

We built a cryogenic-cavity-based ultrastable laser system using this specially designed closed-cycle cryostat. A normal 6 cm sapphire cavity without a vibration-immune design was installed on the cryogenic plate of the cryostat. We locked a 1064 nm laser to the cavity using the Pound–Drever–Hall (PDH) method, and the laser frequency stability was evaluated by beating this laser with another ultrastable reference laser [21,22,23,24]. We obtained the vibrational sensitivity of the cryogenic cavity through different frequencies of vibrational modulation with three voice coil motors driving the cryostat in three perpendicular directions. It is demonstrated that the vibration sensitivity indeed changes as a function of the excitation frequency. The calculated vibrational noise contribution from the measured vibrations and the calibrated vibration sensitivity of the cavity matches well to the measured laser frequency noise. The technique developed paves the way for accurate in situ evaluation of vibrational noise for cryogenic systems, which can be great help for cryostat design and cryogenic precision measurements.

## 2. Vibration Measurement of the Cryostat

Geophone has small size, low cost, and is very sensitive to low audio frequency vibration, but its specified working temperature is only down to around $-40{\;}^{{}^{\circ}}C$. A proper calibration can bring the working temperature of the geophones into the cryogenic temperature [25]. To extend the working temperature of geophones and employ them in vibration measurement at 4 K, we first re-calibrate the geophones, taking into account the induction effect of the vibration sensors at low temperature. The working principle of geophone is based on Faraday’s law: a sensing coil is hung in the casing shield through springs while a fixed magnet is mounted to the bottom of the shield; therefore, the vibrational motion can be sensed when the coil moves with respect to the fixed magnet. The output voltages of the geophone, $V_{1}$, is related to the vibrational motion through velocity sensitivity [25,26]:

(1) $$V_{1}=\left|G{\dot{x}}_{c}\right|=\frac{G\frac{\omega^{2}}{\omega_{0}^{2}}v_{g}}{\sqrt{{\left(1-\frac{\omega^{2}}{\omega_{0}^{2}}\right)}^{2}+4\alpha^{2}\frac{\omega^{2}}{\omega_{0}^{2}}}}.$$

Here, *G* is the velocity sensitivity, ${\dot{x}}_{c}$ is the velocity that the coil moves relative to the magnet, and $v_{g}$ is the measured vibrational motion. ω is the angular frequency, $\omega_{0}=2\pi f_{0}$ is the mechanical resonance frequency. $\alpha =D/2m\omega_{0}$, *D* is the damping coefficient, m=7.51 g is the moving mass of the geophone which can be found in the datasheet. Thus, the voltage output of the geophone can be converted into the velocity, as long as we know the parameters of $\omega_{0}$, *D* and *G*, which can be obtained through a proper calibration procedure. By analyzing the electric circuit, calibration parameters can be fitted from the equation:

(2) $$\left|\frac{V_{1}}{V_{0}}\right|=\sqrt{\frac{{\left(r_{c}D+G^{2}-\omega Lh\right)}^{2}+{\left(\omega Lh+r_{c}h\right)}^{2}}{{\left[\left(R_{s}+r_{c}\right)D+G^{2}-\omega Lh\right]}^{2}+{\left[\left(R_{s}+r_{c}\right)h+\omega Lh\right]}^{2}}},$$

where $V_{0}$ is the source voltage, $r_{c}$ is the coil resistance, $R_{s}$ is the shunt resistance, *L* is the coil inductance, and *h* is a simplified description which is related to the ω and $\omega_{0}$, $h=m\omega (1-\omega_{0}^{2}/\omega^{2})$. At room temperature, ωL is rather small in comparison with $r_{c}$ and, therefore, the coil inductance is normally neglected in the calibration [25,26].

In the calibration, we use a waveform generator to supply an sinusoidal excitation signal with a 0.5 V amplitude and use an oscilloscope to measure the output voltage $V_{1}$ and the source voltage $V_{0}$. The calibration is carried out at the temperatures of 293 and 3.4 K, respectively. The collected voltages $V_{1}$ and $V_{0}$ are fitted from Equation (2), with the parameters $f_{0}$, *G*, $r_{c}$, *D*, and *L* as free parameters to be determined. The fitted parameters averaged from 14 data sets for both cases are summarized in Table 1.

As presented in Table 1, the coils’ resonant frequencies $f_{0}$ at 293 and 3.4 K are fitted to be 9.69±0.35 and 11.16±0.05 Hz, respectively. They are all around 10 Hz and are insensitive to the temperature. Similarly, the velocity sensitivities *G* are almost the same under different temperatures and agree with the specification data. As expected, the coil resistance $r_{c}$ is greatly reduced with decreasing temperature, and the resistance at 3.4 K is calculated to be 0.03±0.04Ω, indicating near-zero coil resistance. The damping coefficient *D* changes from 0.21±0.01 to 0.84±0.01 kg/s due to the reduction of the eddy-current resistance of the coil frame under the cryogenic temperature, which results in an increase of the electromagnetic damping effect [27]. Finally, the inductance at 3.4 K is 0.51±0.01 H, slightly less than the value under 293 K, but the equivalent impedance jωL plays a much more significant role compared with the near-zero coil resistance under cryogenic temperature. Therefore, the inductance effect is not negligible under cryogenic temperature. To be noted is that the geophone sensors we used are designed to be operated in only one orientation, either a horizontal or a vertical direction. The calibration parameters are designed with a difference of less than 10% per specification. In our experiment, we calibrate both directions and find the fitted parameters are in accordance with each other. Therefore, the parameters in Table 1 are fitted from only vertical measurements.

**Figure 1:**
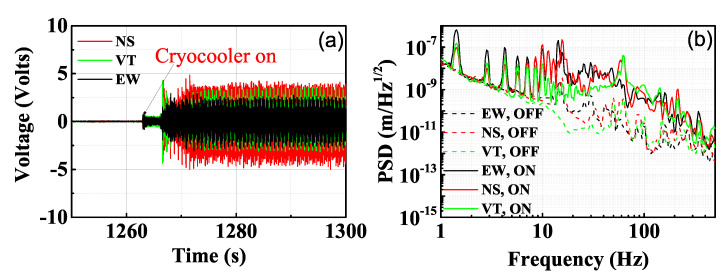
(**a**) Voltage outputs of the geophone sensors when the cryocooler has just been turned on. Vibrations on three independent directions are measured. The red curve for north–south direction (NS), the black curve for east–west direction (EW), and the green curve stands for vertical direction (VT). (**b**) Power spectral density of the vibrational noise on the cryogenic plate. The dashed lines are for measurements when the cryocooler is off and the solid lines are for measurements taken when the cryocooler is on.

We installed three geophone sensors onto the cryogenic plate of a custom-made low-vibration cryostat (Cryomech, PT415) and took measurements while the cryostat was cooling down from room temperature to 3.4 K. The cryostat is designed to be vibration free using liquid helium as a vibration damping agent [28]. The outputs of the geophones are collected by a 24-bit digital acquisition card with a sampling rate of 1000 samples/s. The noise floor of the acquisition system is below $2\times 10^{-8}/f^{2}$$m/{\mathrm{Hz}}^{1/2}$ from 1 to 500 Hz. Figure 1a shows the moment when the cryocooler has just been turned on. Vibrations on three independent directions are measured. The red curve is for north–south direction (NS), the black curve for east–west direction (EW), and the green curve stands for vertical direction (VT), which represents the x, y, z directions in the cryostat’s frame, respectively. The voltage outputs dramatically increase with the turning-on of the pulse-tube cryocooler, indicating much larger vibration levels. Figure 1b shows the vibration power spectral density (PSD) of the cryogenic plate before and after the compressor is turned on under room temperature. The 1.4 Hz resonant frequency of the cryocooler and its harmonics can be clearly seen in the figure, which are caused by the gas pulsation that is generated by the motor and valve rotation. The harmonics are also related to the helium pressure wave that causes periodic stress forces and elastic deformations of the elements of the cryocooler [12,20]. Furthermore, the pulsation and the complicated interaction drive up the vibrational noise level at frequencies higher than 10 Hz. The lowest level of the vibrational noise introduced by the pulsation of the cryocooler can be suppressed when the cryocooler is shut off, shown as the dashed lines in the Figure 1b.

**Figure 2:**
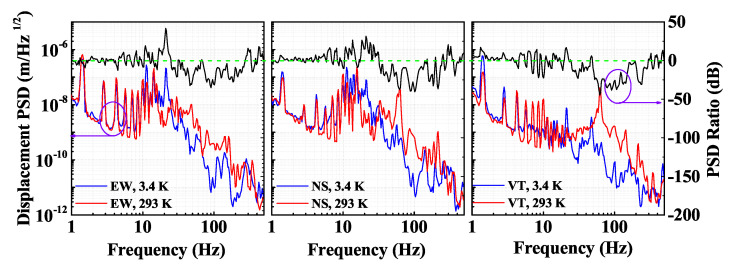
The vibration PSD of the cryogenic plate when the cryocooler has just been turned on at 293 K (red curves) and when it is in stable operation at 3.4 K (blue curves). The upper black curve is the ratio of vibration PSD from 293 to 3.4 K, green dashed line for a 0 dB PSD ratio guide line. Violet circles and arrows are visual guides for the vertical axis.

After cooling down, we measure the vibration level again at 3.4 K, as shown in Figure 2. The line shape is similar to the ones measured under room temperature, but there are several differences. Firstly, the vibration level is suppressed up to 30 dB in all three directions under 3.4 K in comparison with that of 293 K in the 30–500 Hz frequency range. The suppression is caused by the liquid/gas helium mixture that serves as a vibration buffer under cryogenic temperature [28]. Secondly, an extra 20 Hz peak comes out in the 3.4 K vibration spectra, which is not present in the room temperature measurement. It looks like a mechanical resonant frequency due to the introduction of the liquid/gas helium buffer with the reduced temperature. This result shows that it is necessary to use in situ vibration measurement to evaluate the vibration levels under cryogenic temperature. The vibration measurement at room temperature when the cryocooler has just been turned on cannot represent the real vibration levels when the cryocooler is in stable low temperature operation [29]. The vibration noise is two orders of magnitude lower than the reported cryostat vibration which is measured using optical setup [8], and is similar with the vibrational noise measured on an active vibration isolation table with a cryostat on top of it [5]. A clear diagnosis of the vibrational noise of a cryostat is a basic requirement for evaluating the vibrational noise contribution to the cryogenic system, which is essential in many cryogenic precision measurement fields.

## 3. Laser Stabilization and Vibration Noise Evaluation

**Figure 3:**
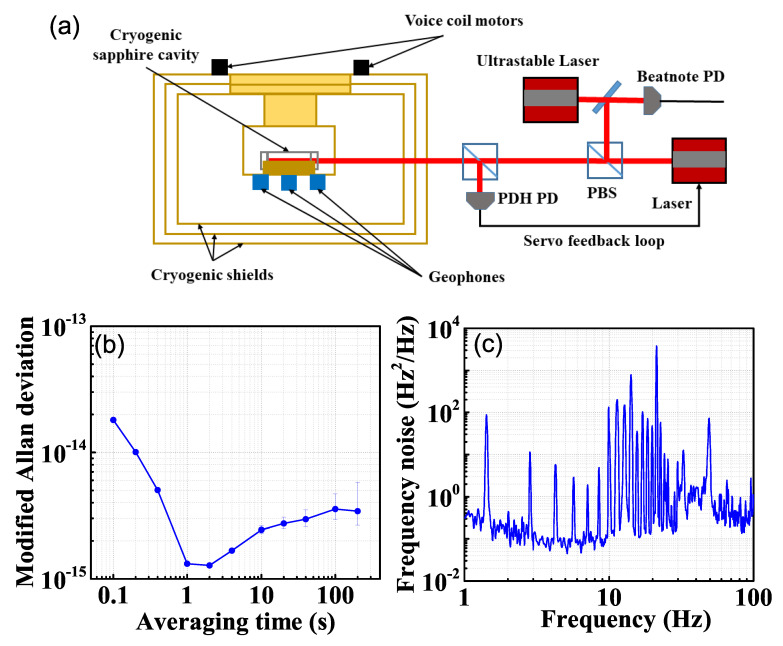
(**a**) Schematic diagram of the laser locking system. (**b**) Cryogenic-sapphire-cavity- based laser stability and (**c**) its frequency noise measured by beating with a room temperature cavity-based laser.

A homemade 6 cm sapphire cavity is placed on an adapter fixed on the cryogenic plate of the closed-cycle cryostat. We lock a 1064 nm continuous wave semiconductor laser to the cavity using the Pound–Drever–Hall (PDH) method [21]. We evaluate the laser frequency stability by beating the laser with another reference ultrastable laser locked to a room temperature cavity [22,23,24], as shown in Figure 3a. The frequency stability of the reference laser is $1.3\times 10^{-16}$ at 1 s averaging time. We downconverted the beat frequency by a signal generator and recorded the frequencies using a frequency counter and a phase noise analyzer. The Allan deviation of the beat frequency is $1.3\times 10^{-15}$ at 1 s averaging time, as shown in Figure 3b. The 1.4 Hz vibrational noise and its harmonics have prominent features in the laser frequency noise spectrum, as shown in Figure 3c. Therefore, a further evaluation of the vibrational noise contribution to laser frequency noise is necessary, which has not been reported in such a hanging-support cryogenic system.

**Figure 4:**
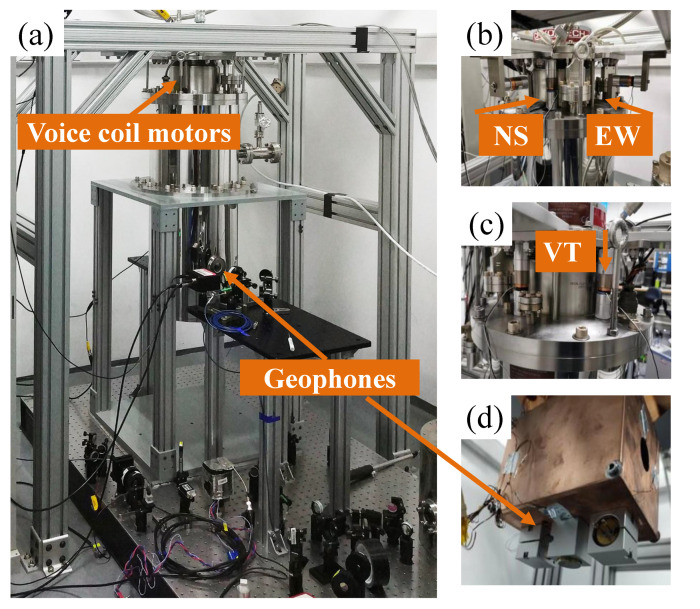
Photo of cryocooler with the vibration excitation and measurement setup. (**a**) The cryocooler. (**b**,**c**) Pictures of voice coil motors on the cryocooler. (**d**) Three geophone sensors for different directions sensing are fixed to the cryogenic plate.

In order to calculate the vibrational noise contribution, we vibrationally excite the system and measure the vibrational sensitivity of the laser system. The vibrational level of the cavity housing can be measured directly by the geophone sensors, as shown in Figure 2. Figure 4 shows the photo of the cryocooler with the vibration excitaion and measurement setup. The photo of the cryocooler is shown in Figure 4A. The mounting stand is bolted to the floor, and the cryogenic chamber is suspended on the mounting stand through the cold head. As shown in Figure 4b,c, voice coil motors are fixed between the mounting stand and the vacuum chamber for three directions vibration excitation. Three geophone sensors are housed in the homemade adapters, as shown in Figure 4d, and fixed to the cryogenic plate that houses the ultrastable cavity. We arrange these voice coil motors symmetrically to balance the excitation force and avoid any tilt.

**Figure 5:**
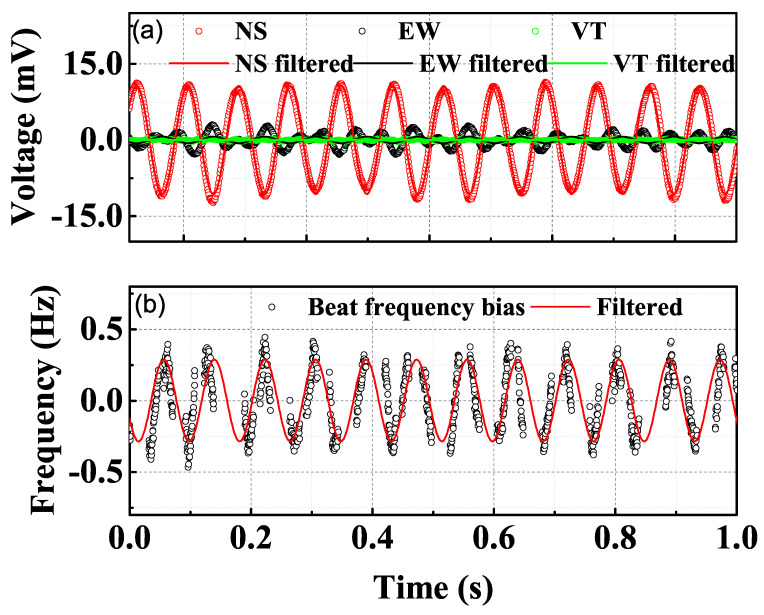
(**a**) Dots are the vibration signal of the cryogenic plate measured by geophones with a 12 Hz vibrational excitation in NS direction, solid curves are the smoothed curves with a 11.5–12.5 Hz bandpass filter. (**b**) Laser frequency bias response to the 12 Hz modulation (dots) and the smoothed curve with the 11.5–12.5 bandpass filter (red curve).

From Figure 3, we can see that the 1.4 Hz vibration and its harmonics are prominent in the laser frequency noise PSD due to the pulse-tube pressure wave. Therefore, we chose several excitation frequencies that do not overlap with the vibrational spikes of the cryostat for the voice coil motors. Figure 5 shows the vibration signal and the laser frequency response to a 12 Hz excitation in NS direction. In the excitation direction, the vibrational noise presents as a robust sinusoidal signal, while there are small couplings in the other two directions. The coupling to the other directions is weak and delayed and, therefore, the vibrations in the coupling directions are normally out of phase with the excitation. We record the laser beating frequency with a 1 kHz sampling rate simultaneously. Both the vibration data and the frequency response data are smoothed with a 11.5–12.5 Hz bandpass filter to remove the distraction of other frequencies. With the vibrational noise data and the frequency response data collected at the same time, we can estimate the vibrational sensitivity of the cavity and evaluate the vibrational contribution to the overall laser frequency noise.

Vibration sensitivity represents how the laser frequency is response to the vibrational noise, and it can be calculated as [30]

(3) $$\Delta \nu_{j}=\sum_{i=1,2,3}a_{ij}k_{i},$$

where *i* is the measured vibrations for the EW, NS, and VT directions, and *j* is the vibration excitation for the three corresponding directions. $a_{ij}$ is the vibration acceleration, $k_{i}$ is the vibrational sensitivity, and $\Delta \nu_{j}$ is the laser frequency fluctuation. With the measured vibration PSD in Figure 2, we can calculate the laser frequency noise due to vibration from Equation (3), where the transfer function can be written as $S_{f_{-}vi}=\sum S_{vi}k_{i}^{2}$, and *i* is for the three directions.

**Figure 6:**
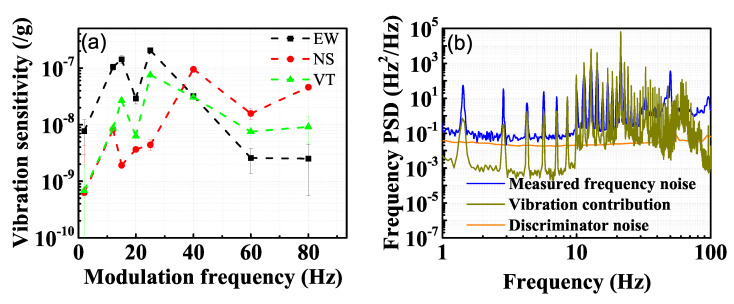
(**a**) Measured vibrational sensitivities for three directions as a function of the excitation frequency. The error bar is enlarged 20 times for viewing purposes. (**b**) The measured frequency noise is shown with the blue curve and the total vibrational noise contribution is shown with the dark yellow curve, which are the measurements of vibrations using the geophones combined with the calibrated vibrational sensitivity of the cavity.

We calculate the vibrational sensitivities of EW, NS, and VT directions by vibration excitation using several excitation frequencies. The results are shown in Figure 6a. Vibrational sensitivities vary from $10^{-10}$ to $10^{-7}$/g level for 10 to 80 Hz excitation. The different vibrational sensitivities of the three directions for different frequency excitation are due to the response of the last supporting stage to the cryostat vibrational noise. All data are calculated from the average of measurements of three groups. The vibrational sensitivities can be separated into two frequency stages, divided at frequency around 50 Hz. When the modulation frequency is lower than 50 Hz, the vibration sensitivities present a ranking as EW larger than VT, while NS is the most insensitive. When the modulation frequency is higher than 50 Hz, the vibration sensitivities present a ranking as NS larger than VT, while EW is the most insensitive. Furthermore, the vibrational sensitivities tend to be damping as the modulation frequency increases.

The total vibrational noise contribution of the three directions using calculated vibrational sensitivities measured by 2 Hz modulation is shown in Figure 6b. By comparing with the measured laser frequency noise, shown with the blue curve, we can see that the vibrational noise is one of the dominant noise of the ultrastable laser system, limited by the large vibrational sensitivity of the cryogenic cavity. The other major noise is the discriminator noise, which is limited by the poor discriminator coefficient of the cavity. The 1.4 Hz vibration resonant frequency of the cryocooler and its harmonics are significant in the measured beat frequency, and the frequencies of the spikes agree well with the calculated vibration contribution. It is noticed that from the vibration contribution spectrum, the harmonic peak is still quite large from 10 to 30 Hz, but the measured frequency spectrum is much smaller. Damping from the mechanical structure is suspected to be responsible for this phenomenon. The envelope around 50 Hz is due to the electric supply. The different sensitivities among different modulation frequencies also results in the difference in the noise spectra.

## 4. Conclusions

In conclusion, we report in situ vibration measurements under cryogenic temperature, and successfully evaluate the vibrational noise contribution to a cryogenic ultrastable system. We measured the vibration levels of the cryogenic plate of a pulse-tube closed-cycle cryocooler under different operation conditions using properly calibrated geophone sensors. The in situ cryogenic vibration measurement right on top of the cryogenic plate clearly demonstrates the vibrational dynamics of the cryostat. The measurement is helpful for understanding the vibrational dynamic inside a cryostat and for better vibration isolation. With real-time and in situ measurement, it also offers the capability to realize vibration feed-forward corrections to suppress the vibrations on the sample area.

We further estimated the vibrational sensitivity of a cryogenic cavity and established the frequency response from the mounting plate down to the vibration excitation. We deduced that a proper vibrational sensitivity calculation cannot rely on a single frequency excitation. This conclusion is insightful for groups working on vibrational noise evaluation of ultrastable cavities. Our result also demonstrates that the our current laser frequency stability of $10^{-15}$ is partly limited by the vibrational noise due to the non-vibration-insensitive design of the cavity and, thus, the cryogenic ultrastable laser system still struggles with vibrational noise. Much better performance is expected with a vibration-insensitive cavity design. A 100-fold improvement in the vibrational sensitivity, which has been reported by other groups, can readily push the vibrational noise contribution to laser stability to the $10^{-17}$ level [3].

## Figures and Tables

**Table 1:** Parameters fitted from Equation (2), including room temperature calibration and cryogenic temperature calibration. The error bars of the data are calculated from 14 data sets.

Parameter	Specification@25 ${}^{{}^{\circ}}$C	Fitted@293 K	Fitted@3.4 K
$f_{0}$(Hz)	10	9.69±0.35	11.16±0.05
*G*(V/m/s)	37.8	37.5±0.36	36.6±0.24
$r_{c}$(Ω)	1250	1160±12	0.03±0.04
*D*(kg/s)	0.25	0.21±0.01	0.84±0.01
*L*(H)	0.3	0.61±0.02	0.51±0.01

## Data Availability

The data that support the findings of this study are available within the article.
